# A Manual of Midwifery for Midwives

**Published:** 1883-12

**Authors:** 


*A Manual of Midwifery for Midwives.* By FANCOURT BARNES, M.D., M.R.C.P. Second Edition. Pp. 181. London: Smith, Elder, & Co. 1883.

The second edition of this little work has been improved by several corrections and additions. We think that future editions might be made more useful to midwives by a judicious pruning of the purely scientific part of the work, and a fuller and more exact and detailed account of their purely practical duties. As it exists, it is undoubtedly well put together, and will "supply a felt want."

